# Intensity-modulated radiation therapy versus three-dimensional conformal radiotherapy in head and neck squamous cell carcinoma: long-term and mature outcomes of a prospective randomized trial

**DOI:** 10.1186/s13014-020-01666-5

**Published:** 2020-09-16

**Authors:** Tejpal Gupta, Shwetabh Sinha, Sarbani Ghosh-Laskar, Ashwini Budrukkar, Naveen Mummudi, Monali Swain, Reena Phurailatpam, Kumar Prabhash, Jai Prakash Agarwal

**Affiliations:** 1 Department of Radiation Oncology, Tata Memorial Hospital (TMH)/Advanced Centre for Treatment Education & Research in Cancer (ACTREC), Tata Memorial Centre, Homi Bhabha National Institute (HBNI), Kharghar, Mumbai, 410210 India; 2 Department of Medical Physics, Tata Memorial Hospital (TMH)/Advanced Centre for Treatment Education & Research in Cancer (ACTREC), Tata Memorial Centre, Homi Bhabha National Institute (HBNI), Mumbai, India; 3 Department of Medical Oncology, Tata Memorial Hospital (TMH)/Advanced Centre for Treatment Education & Research in Cancer (ACTREC), Tata Memorial Centre, Homi Bhabha National Institute (HBNI), Mumbai, India

**Keywords:** Head-neck cancer, Outcomes, Radiotherapy, Subcutaneous fibrosis, Xerostomia

## Abstract

**Purpose:**

To compare long-term disease-related outcomes and late radiation morbidity between intensity-modulated radiation therapy (IMRT) and three-dimensional conformal radiotherapy (3D-CRT) in head and neck squamous cell carcinoma (HNSCC) in the setting of a prospective randomized controlled trial.

**Methods:**

Previously untreated patients with early to moderately advanced non-metastatic squamous carcinoma of the oropharynx, larynx, or hypopharynx (T1-T3, N0-N2b, M0) planned for comprehensive irradiation of primary site and bilateral neck nodes were randomly assigned to either IMRT or 3D-CRT after written informed consent. Patients were treated with 6MV photons to a total dose of 70Gy/35 fractions over 7 weeks (3D-CRT) or 66Gy/30 fractions over 6 weeks (IMRT). A sample size of 60 patients was estimated to demonstrate 35% absolute difference in the incidence of ≥grade 2 acute xerostomia between the two arms. All time-to-event outcomes were calculated from date of randomization until the defined event using the Kaplan-Meier method.

**Results:**

At a median follow-up of 140 months for surviving patients, 10-year Kaplan-Meier estimates of loco-regional control (LRC); progression-free survival (PFS); and overall survival (OS) with 95% confidence interval (95%CI) were 73.6% (95%CI: 61.2–86%); 45.2% (95%CI: 32–58.4%); and 50.3% (95%CI: 37.1–63.5%) respectively. There were no significant differences in 10-year disease-related outcomes between 3D-CRT and IMRT for LRC [79.2% (95%CI: 62.2–96.2%) vs 68.7% (95%CI: 51.1–86.3%), *p* = 0.39]; PFS [41.3% (95%CI: 22.3–60.3%) vs 48.6% (95%CI: 30.6–66.6%), *p* = 0.59]; or OS [44.9% (95%CI: 25.7–64.1%) vs 55.0% (95%CI: 37–73%), *p* = 0.49]. Significantly lesser proportion of patients in the IMRT arm experienced ≥grade 2 late xerostomia and subcutaneous fibrosis at all time-points. However, at longer follow-up, fewer patients remained evaluable for late radiation toxicity reducing statistical power and precision.

**Conclusions:**

IMRT provides a clinically meaningful and sustained reduction in the incidence of moderate to severe xerostomia and subcutaneous fibrosis compared to 3D-CRT without compromising disease-related outcomes in long-term survivors of non-nasopharyngeal HNSCC.

## Introduction

The contemporary standard of care in non-surgical, curative-intent management of head and neck squamous cell carcinoma (HNSCC) is definitive radiotherapy (RT) with or without concurrent chemotherapy [1, 2]. Traditionally, HNSCC had been treated with conventional RT techniques which generally comprised of simple field arrangements, typically parallel-opposed portals with or without matching low anterior neck field or antero-lateral wedge pair based on two-dimensional (2D) fluoroscopic imaging with no major emphasis on shielding of normal tissues. Such conventional techniques led to considerable morbidity [3] such as dryness of mouth, sticky saliva, swallowing dysfunction, and subcutaneous fibrosis with resultant negative impact upon health-related quality-of-life (QOL) in long-term survivors [4, 5]. Over the years, technological advances in treatment planning and delivery based on computed tomographic (CT) imaging have resulted in progressive conformation of radiation dose to the target tissues while sparing adjacent organs-at-risk (OARs) ushering in the era of three-dimensional conformal RT (3D-CRT). In the last decade or so, intensity-modulated radiation therapy (IMRT), an advanced form of high-precision conformal RT that uses non-uniform beam intensities calculated through computer-controlled optimization to achieve the desired dose-distribution has largely supplanted older radiation techniques (2D-RT/3D-CRT) based on consistent high-quality evidence demonstrating significant reduction in radiation-induced xerostomia [6, 7]. The time-frame at which radiation-induced xerostomia outcomes have been reported in randomized controlled trials (RCTs) has been very variable [8–14] ranging from acute xerostomia (within 3-months) to delayed xerostomia (typically within 1–3 years of completion of therapy). It is widely accepted that salivary gland function starts recovering 3–6 months after RT and gradually improves over time [3, 6, 7]; hence, whether the difference in IMRT versus conventional 2D-RT/3D-CRT persists at a longer follow-up beyond 5-years is largely unknown. We had earlier reported the safety and efficacy outcomes [11] as well as dose-response relationship of the parotid glands [15] from our randomized trial comparing IMRT with 3D-RT in the curative-intent radiotherapeutic management of non-nasopharyngeal HNSCC in the definitive setting. Herein, we report and compare the long-term disease-related outcomes and late toxicity of the index trial at an extended and mature follow-up (> 10 years).

## Aims & objectives

The main objective of the trial was to demonstrate the superiority of parotid-sparing IMRT over 3D-CRT using the incidence of physician-rated acute salivary gland toxicity (≥grade 2) as the primary endpoint. Secondary endpoints included other acute toxicity (mucositis, dermatitis, dysphagia), late radiation morbidity, patterns of failure, loco-regional disease status, and overall survival.

## Materials and methods

### Design and eligibility

Trial design, eligibility, and conduct have been described in detail previously [11, 15]. Briefly, biopsy-proven and previously untreated patients with early to moderately advanced non-metastatic squamous carcinoma of the oropharynx, larynx (excluding T1 glottic cancer), or hypopharynx (T1-T3, N0-N2b, M0) as per Tumor-Node-Metastases (TNM) classification of the 7th edition of American Joint Committee on Cancer (AJCC) staging requiring comprehensive irradiation of primary tumor and bilateral neck nodes were included. After written informed consent, patients were randomly assigned in a 1:1 ratio to either IMRT or 3D-CRT using computer-generated permuted-block design with stratification for T-stage (T1–2 vs T3), N-stage (N0–1 vs N2), and site of primary (oropharynx vs hypopharynx vs larynx). Patients underwent salivary scintigraphy and appropriate dental prophylaxis at baseline (prior to treatment) and longitudinally on follow-up at pre-specified time-points. The trial was duly approved in 2004 by the Institutional Review Board that functions in accordance with the Declaration of Helsinki and completed accrual in April 2008 ensuring a minimum follow-up of over 10-years for surviving patients. The trial is registered with Clinical Trials Registry of India (CTRI) and was partially funded through an academic research grant by an industry vendor to the institute. However, the funding source was not involved in study design, conduct, analysis, or interpretation. Trial records were vested with the Principal Investigator and corresponding author of this report, who takes full public responsibility for integrity and authenticity of submitted data.

### Treatment planning and delivery

Details of treatment planning and delivery have been published previously [11]. Briefly, 3D-CRT was planned and delivered in 2–3 sequential phases summated to get the composite treatment plan. First phase of 3D-CRT was planned with 6 MV photons using 7–9 multi-leaf collimator (MLC) shaped coplanar beams with wedges, weightage, and compensative fields as appropriate. This was followed by sequential boost plan(s) with simpler beam geometry (3–4 conformal fields) for a total tumor dose of 70Gy/35 fractions over 7 weeks. IMRT planning was done with 6MV photons with 7–9 equispaced coplanar beams using the simultaneous integrated boost (SIB) technique to a total tumor dose of 66Gy/30 fractions over 6 weeks to the high-risk target volumes with the intermediate-risk and low-risk elective volumes receiving 60Gy/30 fractions and 54Gy/30 fractions respectively over 6 weeks. The planning objective for IMRT was to restrict doses to the contralateral parotid gland (mean dose ≤26Gy) and spinal cord (maximum dose <45Gy), while ensuring that ≥95% of the target volume was covered by at least 95% of the prescribed dose. None of the patients in the study received induction chemotherapy. Patients with advanced stage disease (bulky T2, T3 and/or node positive) with adequate renal function (defined as creatinine clearance > 50 ml/min) received concurrent weekly low-dose cisplatin (30 mg/m^2^) with appropriate anti-emetic prophylaxis, adequate hydration and forced saline diuresis as per institutional protocol.

### Follow-up assessments

Patients were scheduled for response assessment 18F-flouro-deoxy-glucose positron emission tomography (FDG-PET)/CT at 8–12 weeks after completion of therapy. Patients with complete morphological and metabolic response at primary site and neck on FDG-PET/CT were followed-up clinically every 3-monthly for the first 2 years, 6-monthly until 5 years, and annually thereafter. Only patients with abnormal focal FDG-uptake in the node(s) and/or residual palpable node (persistent disease) were considered for neck dissection, provided the tumor at primary site was adequately controlled. The trial was originally designed for 5-year follow-up period and longer intervals (18–24 months) between follow-ups were also acceptable based on patient’s request beyond 5 years post-treatment. Patients were assessed periodically for radiation morbidity both clinically using the Radiation Therapy Oncology Group (RTOG) toxicity scoring criteria as well as via investigations such as salivary scintigraphy [15], pure-tone audiometry, and blood investigations including thyroid function tests. Surveillance imaging was not routinely performed but restricted to patients presenting with new-onset symptoms or suspected recurrence on clinical examination. Salvage surgery for local and/or regional recurrence was considered for selected cases in either arm after discussion in a multi-disciplinary head and neck tumor board.

### Statistical analyses

Based on the premise of 35% absolute difference in the incidence of ≥grade 2 acute salivary gland toxicity between 3D-CRT (85%) and IMRT (50%), a sample size of 54 patients was estimated using an ‘α’ error of 0.05 and a ‘β’ error 0.20 (one-tailed test of significance). Accounting for 10% non-evaluable patients (lost to follow-up or assessments not done) for the primary endpoint, a total of 60 patients were required to be randomized. Chi-squared test was used to demonstrate the difference in proportion of patients with grade 2 or worse toxicity (xerostomia and subcutaneous fibrosis) between the two arms at various pre-specified time-points. Any persistent, residual, or recurrent disease at the primary site or neck was considered an event for loco-regional control (LRC), without taking salvage surgery into account. In addition to the events for loco-regional failure, distant metastases and/or death were considered as events for progression-free survival (PFS). Death from any cause was considered as event for overall survival (OS). For all time-to-event analyses, calculation was done from date of randomization until the defined event using the product-limit method of Kaplan-Meier and compared using the log-rank test. The cut-off date for analysis was 31st October 2019. All statistical analyses were done on SPSS version 24.0. Given the limited number of patients included with further attrition on long-term follow-up (> 5-years), our study was not adequately powered to reliably detect statistically significant differences between the two arms for either efficacy or late toxicity-related endpoints.

## Results

Patient demographics and study flow have been described in detail previously [11]. Overall, the study cohort was largely representative of the typical head-neck cancer population in the community with no significant differences in the baseline characteristics between the two arms. Notably, oropharynx was the site of primary in > 50% of patients in both arms; however, human papilloma virus (HPV) testing was not done routinely in the study, as it had not been recognized as a distinct clinical entity at the time of trial design and accrual. Patients with laryngeal and hypopharyngeal cancers was also equitably distributed in the two arms [11]. None of the patients had hyposalivation prior to treatment as seen on the baseline salivary scintigraphy scan [15]. Expectedly, IMRT resulted in significant reduction in mean doses to both parotid glands compared to 3D-CRT. As reported previously [11], the mean dose with 95% confidence intervals (95%CI) to the contralateral parotid gland was 49.8Gy (46.5–53.1Gy) and 28.8Gy (27–30.7Gy) in the 3D-CRT and IMRT arms respectively (*p* < 0.0001). Similarly, mean dose (95%CI) to the ipsilateral parotid gland was also consistently and significantly lesser at 39.8Gy (36.3–43.2Gy) with IMRT compared to 56.2Gy (52.5–60.1Gy) with 3D-CRT (*p* < 0.0001). There were no statistically significant differences between other acute toxicities (mucositis, dermatitis, dysphagia and weight loss) between the two arms [11]. Toxicity and disease-related outcomes have been previously reported at a median follow-up of 40 months with an inter-quartile range (IQR) of 26–50 months [11]. Presently, the outcomes are being reported at a median follow-up of 140 months (IQR = 129–147.5 months) for surviving patients.

### Disease-related outcomes

**Fig. 1:**
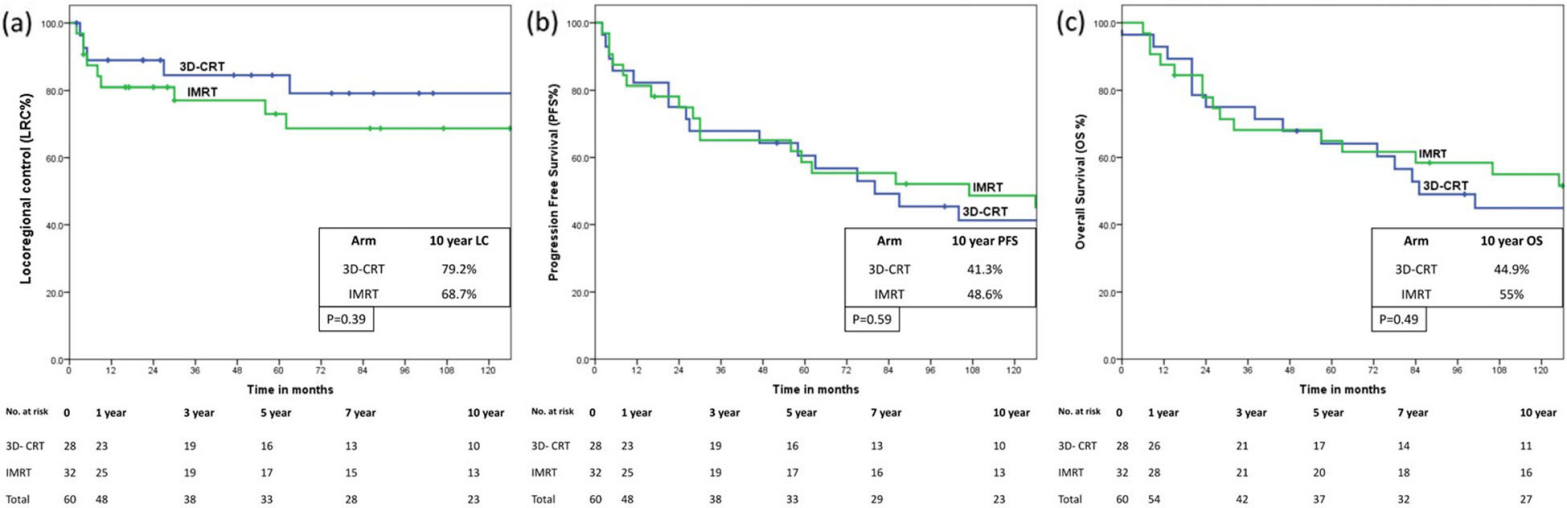
Kaplan-Meier estimates showing no significant difference between three-dimensional conformal radiotherapy (3D-CRT) versus intensity modulated radiation therapy (IMRT) for 10-year loco-regional control (**a**); progression-free survival (**b**), and overall survival (**c**) in patients with early to moderately advanced non-nasopharyngeal head and neck cancers

The patterns of the first failure and distribution by treatment arm is summarized in Table 1. As of last follow-up, 18 of 28 (64.2%) and 18 of 32 (56.3%) patients had not experienced any disease-related events in the 3D-CRT and IMRT arms respectively. Two patients (both in the IMRT arm) did not have any residual viable tumor on neck dissection for persistent residual palpably enlarged node and were not considered as having disease-related events. Notably, the response assessment FDG-PET/CT was not showing any increased uptake in the residual palpable node in both cases. Additionally, one patient each in both arms underwent successful salvage neck dissection for regional recurrence and remained loco-regionally controlled till last follow-up. A total of 9 (15%) patients [4 of 28 (14.2%) in 3D-CRT arm and 5 of 32 (15.7%) in IMRT arm] developed another new primary cancer (Table 2) resulting in 8 deaths, while one patient was successfully salvaged by wide local excision of a superficially invasive squamous carcinoma of the buccal mucosa in the 3D-CRT arm. By the time of this analysis, 31 patients have died, 27 patients are alive [11 of 28 (39.8%) in 3D-CRT arm and 16 of 32 (50%) in IMRT arm], while 2 patients (one in each arm) are lost to follow-up. As expected, several patients succumbed to comorbidities unrelated to the index cancer. Non-cancer related deaths were documented in 11 of 60 (18.3%) patients [8 of 28 (28.5%) in the 3D-CRT arm vs 3 of 32 (9.3%) in the IMRT arm]. The 10-year Kaplan-Meier estimate of LRC was 73.6% (95%CI: 61.2–86%) for the entire study cohort without accounting for salvage surgery. Similar estimates of PFS and OS were 45.2% (95% CI: 32.8.4%) and 50.3% (95%CI: 37.1–63.5%) respectively for the entire study cohort. There were no significant differences in 10-year disease-related outcomes between 3D-CRT and IMRT for either LRC [79.2% (95%CI: 62.2–96.2%) vs 68.7% (95%CI: 51.1–86.3%), *p* = 0.39]; PFS [41.3% (95%CI: 22.3–60.3%) vs 48.6% (95%CI: 30.6–66.6%), *p* = 0.59]; or OS [44.9% (95%CI: 25.7–64.1%) vs 55.0% (95%CI: 37–73%), *p* = 0.49] respectively (Fig. 1).

**Table 1:** Patterns of first failure by treatment arm in the study cohort (*N* = 60)

Disease-related event(s) of interest	3D-CRT^**a**^ (%) (***n*** = 28)	IMRT^**b**^ (%) (***n*** = 32)
No documented disease-related event	18 (64.2%)	18 (56.3%)
Persistent primary site (local) disease	1 (3.6%)	1 (3.1%)
Persistent neck nodal (regional) disease	1 (3.6%)	0 (0%)
Primary site (local) recurrence	0 (0%)	2 (6.2%)
Neck nodal (regional) recurrence	1 (3.6%)	1 (3.1%)
Primary + nodal (loco-regional) recurrence	1 (3.6%)	2 (6.2%)
Isolated distant metastases	1 (3.6%)	0 (0%)
Distant + primary + nodal failure	1 (3.6%)	3 (9.4%)
Second new primary	4 (14.2%)	5 (15.7%)

^a^ *3D-CRT* three-dimensional conformal radiotherapy, ^b^ *IMRT* intensity-modulated radiation therapy

**Table 2:** Site of second new primary, time-interval from index cancer and final clinical outcomes

Sr. No.	Arm	Primary site	Site of second new primary cancer	Time-interval from index cancer	Final clinical outcome
1	3D-CRT^a^	Base of tongue	Buccal mucosa	13 months	Alive without disease
2	3D-CRT	Tonsil	Alveolus	72 months	Died of post-operative complications
3	3D-CRT	Pyriform sinus	Esophagus	19 months	Died of 2nd malignancy
4	3D-CRT	Glottic larynx	Esophagus	100 months	Died of 2nd malignancy
5	IMRT^b^	Tonsil	Lung	19 months	Died of 2nd malignancy
6	IMRT	Pyriform sinus	Lung	27 months	Died of 2nd malignancy
7	IMRT	Supraglottic larynx	Urinary bladder	64 months	Cured of bladder cancer
			Tonsil (3rd primary)	72 months	Died of 3rd malignancy
8	IMRT	Base of tongue	Pyriform sinus	95 months	Died of 2nd malignancy
9	IMRT	Tonsil	Esophagus	56 months	Died of 2nd malignancy

^a^*3D-CRT* three-dimensional conformal radiotherapy, ^b^ *IMRT* intensity-modulated radiation therapy

### Late toxicity

**Fig. 2:**
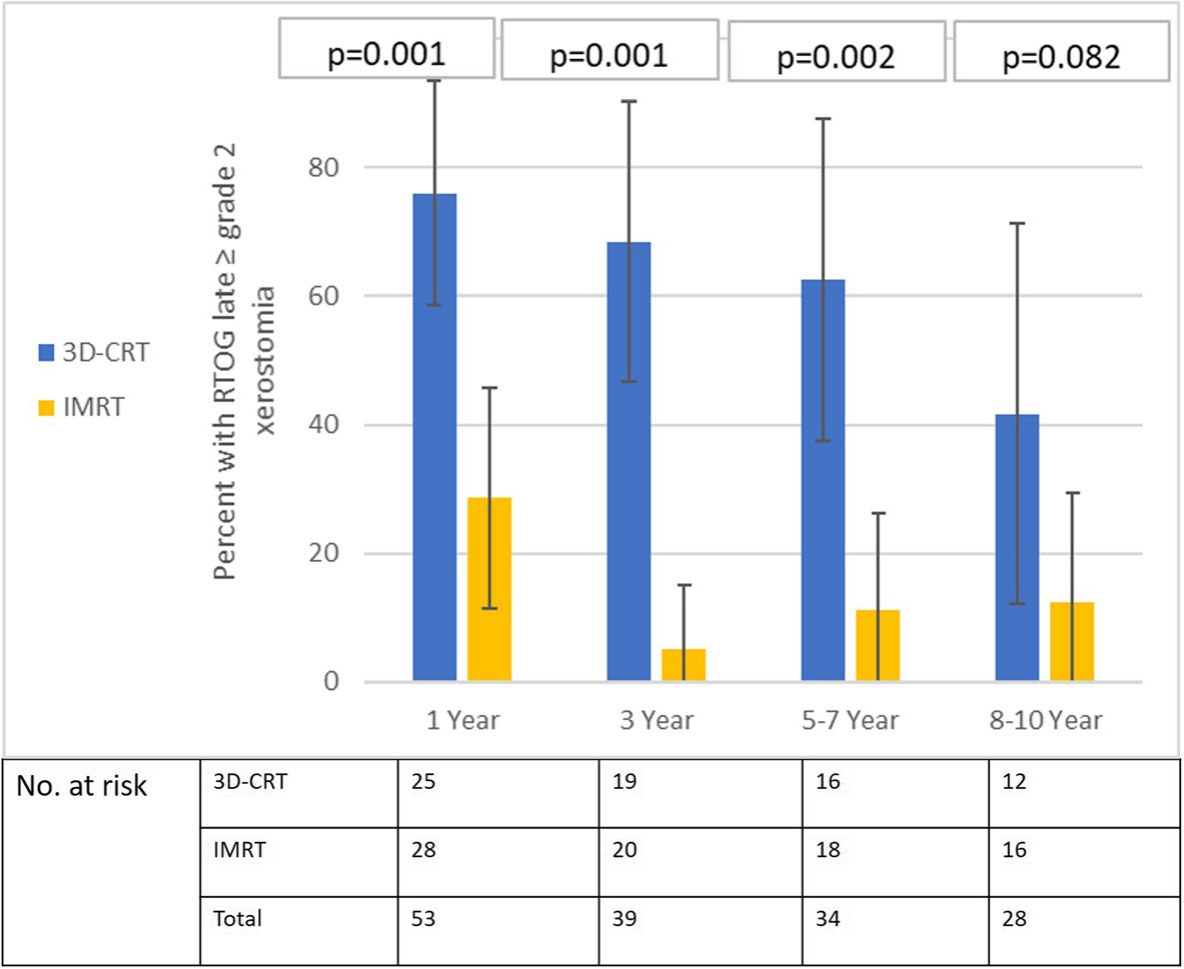
Proportion of patients (error-bars represent 95% confidence intervals) with moderate to severe (≥grade 2) late xerostomia at specified time-points in three-dimensional conformal radiotherapy (3D-CRT) and intensity modulated radiation therapy (IMRT) arms. Note the statistically significant *p*-values favouring IMRT consistently. Lesser number of patients at risk in both arms on long-term follow-up (at 8–10 years) reduces statistical power but, clinically meaningful difference is sustained over time

**Fig. 3:**
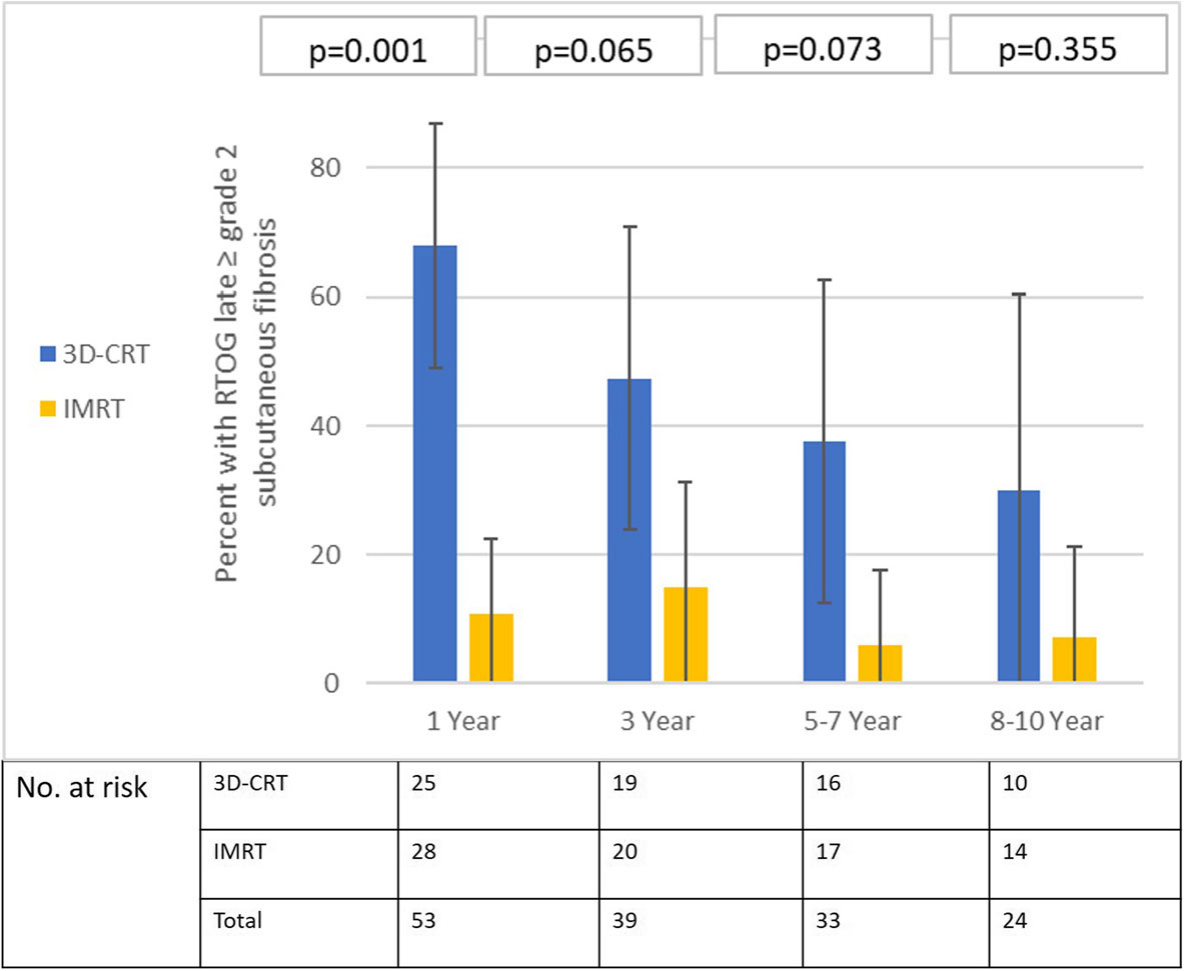
Proportion of patients (error-bars represent 95% confidence intervals) with moderate to severe (≥grade 2) subcutaneous fibrosis at specified time-points in three-dimensional conformal radiotherapy (3D-CRT) and intensity modulated radiation therapy (IMRT) arms. Note the statistically significant p-values favouring IMRT in the medium-term (1 and 3 years). Lesser number of patients at risk in both arms on long-term follow-up (between 5 and 10 years) reduces statistical power, but, clinically meaningful difference persists with time

The proportion (95%CI) of patients with physician-defined ≥grade 2 salivary gland toxicity by arm is plotted longitudinally over time in Fig. 2. As expected, significantly lesser proportion of patients in the IMRT arm experienced RTOG grade 2 or worse salivary toxicity at all specified time-points. However, at longer follow-up (> 8–10 years), lesser number of patients remained at risk and evaluable for late toxicity reducing statistical power and precision. At around 10 years, ≥grade 2 xerostomia was seen in 5 of 12 patients (41.7, 95%CI: 29.6–41.7%) in the 3D-CRT arm compared to 2 of 16 (12.5, 95%CI: 0–29.5%) in the IMRT arm, a clinically meaningful difference, though of borderline statistical significance (*p* = 0.082). Four patients in 3D-CRT arm had late grade 3 xerostomia while no patient in IMRT experienced such severe xerostomia. The proportion (95%CI) of patients with moderate to severe subcutaneous fibrosis by arm over time is presented in Fig. 3. Similar to xerostomia, lesser proportion of patients in the IMRT arm developed grade 2 or worse late subcutaneous fibrosis at all specified time-points. At 10 years or so, ≥grade 2 subcutaneous fibrosis was documented in 3 of 10 (30, 95%CI: 0–60.4%) in the 3D-CRT arm compared to 1 of 14 (7.1, 95%CI: 0–21.3%) in the IMRT arm, again a clinically meaningful difference, though statistically not significant (*p* = 0.35). In the long-term, there was no significant difference in incidence of radiation-induced hypothyroidism between the two arms [5 of 28 (17.9%) in 3D-CRT arm vs 7 of 32 (21.9%) in IMRT arm, *p* = 0.79]. The incidence of other late toxicities was too small for any valid statistical comparison. Other significant late toxicities included 3 cerebrovascular accidents (all in the 3D-CRT arm), 1 pharyngeal stricture (3D-CRT arm) requiring repeated dilatation and 1 case of suspected chondroradionecrosis (IMRT arm) which was salvaged with aggressive supportive care. Significant hoarseness of voice was reported by 2 patients (1 in each arm). None of the surviving patients in either arm had feeding tube dependence or dysfunctional larynx on long-term follow-up.

## Discussion

To the best of our knowledge, this is the first long-term and mature (at 10-years) report of clinical outcomes of any RCT comparing IMRT with older techniques in the indexed medical literature. To date, seven RCTs involving 1155 patients have directly compared IMRT with 2D-RT (3 trials) or 3D-CRT (4 trials) in HNSCC [8–14]. Four such trials included patients with non-nasopharyngeal HNSCC (oropharyneal, laryngo-pharyngeal, and oral cavity primaries), while the other three were limited to nasopharyngeal cancers. Five of them were rather small comprising fewer than 100 patients (in both arms), with only one trial having a large sample size (> 600 patients). The primary objective in 5 index RCTs was salivary gland toxicity (xerostomia), with only 1 trial each using LRC and OS as primary endpoints respectively for sample size calculation. All of them reported early salivary gland toxicity, but only three RCTs reported xerostomia at 3-years [11, 13, 14] with > 5-year xerostomia outcomes being reported in only a single trial [13]. The mean absolute difference in the proportion of patients with moderate to severe xerostomia at 1-year between 3D-CRT and IMRT was around 23% but varied widely across different studies ranging from 14% (24% vs 10%) in one Indian study [13] to 47% (66 to 19%) in the French study [14] in favour of IMRT. Pooling of data from all seven RCTs for quantitative synthesis in a meta-analysis [7] demonstrated that the use of IMRT was associated with a significant reduction in relative risk (RR) of acute ≥grade 2 xerostomia (RR = 0.64, 95%CI = 0.49–0.84; *p* = 0.001) compared to 2D-RT/3D-CRT. Furthermore, significantly reduced risk of grade 2 or worse xerostomia with IMRT was seen at all time-points (6-months, 1-year, 2-years, and 3-years post-treatment). The use of IMRT was also associated with a relative reduction in the risk of loco-regional recurrence with a hazard ratio (HR) of 0.76 (95%CI: 0.57–1.01; *p* = 0.06) and relative reduction in risk of death (HR = 0.70, 95%CI = 0.57–0.88; *p* = 0.002) compared to 2D-RT/3D-CRT, albeit with low statistical power due to inadequate patient numbers. However, this benefit of IMRT for LRC and OS was restricted to nasopharyngeal cancer, largely driven by the large Chinese study [12], with no significant difference in efficacy between the two techniques in patients with cancers of the oropharynx and laryngo-pharynx. QOL outcomes could not be pooled in the meta-analysis due to inadequate and incomplete reporting of data.

One of the most common and debilitating toxicity of head-neck irradiation is xerostomia (subjective sensation of a dry mouth) caused by salivary gland hypofunction (decrease in salivary flow or output) leading to persistent dryness of mouth, sticky saliva, oral discomfort, and difficulty in speech and swallowing with consistent negative impact upon health-related QOL [3, 5]. It is widely believed that salivary function does recover over time [6, 7, 16] with demonstrable improvement in subjective symptoms of xerostomia (dryness of mouth and excessive thirst) largely due to compensatory increase in acinar cell production although these new acinar cells are thought to have a different morphology than the unirradiated ones [17]. In the current report, we have demonstrated that the clinically meaningful benefit of parotid-sparing IMRT over 3D-CRT in reducing moderate to severe xerostomia is sustained over time even at 8–10-years after treatment. Grade 3 late xerostomia, which can be severely debilitating was not seen in any patient treated with IMRT, but, was documented in 4 patients (3 dead and 1 alive without disease) in the 3D-CRT arm. The sustained long-term benefit with IMRT may be attributable in part to the dose-volume histogram patterns in salivary gland sub-volumes [18] including lesser doses of irradiation to parotid stem cells (compared to conventional techniques) with potential to influence salivary injury and recovery leading to better post-treatment regenerative capacity and gradual progressive recovery of salivary function. In keeping with the prevailing guidelines and recommendations at that time [19], we had mandated a mean dose of ≤26Gy as the dose-volume constraint for the contralateral parotid gland. This is much higher than the current Quantifying Normal Tissue Effects in the Clinic (QUANTEC) guidelines [20] that recommend keeping the mean dose for single parotid gland to below 20Gy during optimization to reduce the risk of moderate to severe xerostomia.

Apart from xerostomia, we also demonstrated significant reduction in the risk of moderate to severe subcutaneous fibrosis with IMRT compared to 3D-CRT that was also largely sustained over time. Although, dose-constraints were not applied separately, reduction in doses to subcutaneous tissue with IMRT may have led to better long-term restoration of vascularity in the dermal and subdermal layers. Mean doses to the thyroid gland were similar in both arms (dose-volume constraints not applied separately) with no significant difference in the incidence of biochemical hypothyroidism between the two techniques. The incidence of other late toxicities was too small for any meaningful statistical comparison. Three cases of cerebrovascular accidents (all in 3D-CRT arm) may have been induced by high doses of RT to bilateral carotid arteries with resultant stenosis and compromised vascular supply to the brain [21]. Non-cancer related deaths were somewhat higher in the 3D-CRT arm including stroke and aspiration pneumonia, although the exact cause of death was not known in four patients. As head and neck cancer survivorship improves, consensus guidelines and newer dose-volume constraints for various other OARs such as dysphagia-aspiration related structures (DARS) and carotid arteries would need to be tested in prospective studies to reduce some of the late morbidities and resultant non-cancer related deaths [22, 23]. The incidence of second new primaries was quite similar in both the arms of our study raising doubts over the hypothesis that IMRT is associated with an increased incidence of second malignant neoplasms due to larger volumes of low-dose spillage and resultant higher integral doses [24].

Some previous studies [25, 26] of IMRT have shown marginal recurrence rates of 5–15% in the vicinity of the spared parotid gland raising valid concerns regarding the safety of such an approach. Reassuringly, the long-term rates of disease-related outcomes (LRC and OS) were quite similar in both arms of our study suggesting that parotid-sparing was not at the expense of disease control. We followed standard target volume delineation and contouring guidelines with stringent quality control in treatment planning and delivery to ensure the safety of IMRT. However, our study was not adequately powered to demonstrate equivalence or non-inferiority of IMRT over 3D-CRT in terms of disease-related outcomes (LRC or OS), which would need over a thousand patients to be randomized.

### Caveats and limitations

Despite the strength of a prospective RCT with long-term and mature follow-up, certain caveats and limitations remain. Given the difference in RT techniques, we could not blind patients or physicians to treatment arm leaving room for ascertainment and reporting bias. The use of different dose and dose fractionation in the two arms, though deemed radiobiologically equivalent, could also be a potential source of bias. The number of patients included and randomized on our study was quite small (*N* = 60), with even much lesser numbers on long-term follow-up (between 5 and 10 years), impairing statistical power, precision, and validity of the late toxicity analyses. We tried to spare only the contralateral parotid gland without attempting submandibular gland sparing [27], which is the greatest contributor to whole saliva during rest and is a better moistener for oral tissues. Underestimation of xerostomia cannot be entirely ruled out as we used physician-rated xerostomia as the primary endpoint and not patient-reported outcomes. A xerostomia-specific questionnaire was not used in our study which would have been more useful rather than a general QOL instrument. We did not test our patients with p16 immunohistochemistry to identify HPV-associated oropharyngeal cancer which is now established as a distinct clinical entity with prognostic implications and a separate new staging system [28]. Apart from increasing availability of particle beam therapy with its unique depth-dose characteristics [29], the last decade has also witnessed further technological improvements in photon-based treatment planning, delivery, and verification [30] with the introduction of volumetric modulated arc therapy/rotational IMRT, robust optimization, in-room image-guidance, and adaptive RT, none of which was used in our study.

## Conclusions

This report provides the best available evidence for a sustained clinically meaningful benefit of IMRT compared to 3D-CRT in reducing the late morbidity of radiation (moderate to severe xerostomia and subcutaneous fibrosis) without compromising disease-related outcomes in long-term survivors of non-nasopharyngeal HNSCC. A similar risk of second malignant neoplasms and apparent decrease in non-cancer related deaths provides further compelling arguments towards adopting IMRT as the contemporary standard of care in the radiotherapeutic management of patients with HNSCC.

## Supplementary information

**Additional file 1 Supplementary Fig. 1**

## Data Availability

The datasets generated and/or analysed during the current study are not publicly available due to institutional norms but are available from the corresponding author on reasonable request.

## Abbreviations

IMRT: Intensity Modulated Radiotherapy
3D-CRT: Three dimensional Conformal Radiotherapy
HNSCC: Head Neck Squamous Cell Carcinoma
LRC: Loco-Regional Control
PFS: Progression Free Survival
OS: Overall Survival
RT: Radiotherapy
CT: Computerized Tomography
OAR: Organs at Risk
RCT: Randomized Controlled Trial
TNM: Tumor Node Metastasis
AJCC: American Joint Committee on Cancer
FDG PET-CT: Fluorodeoxyglucose (FDG)-Positron Emission Tomography (PET) Computerized Tomography
RTOG: Radiation Therapy Oncology Group
HPV: Human Papilloma Virus
QUANTEC: Quantitative Analyses of Normal Tissue Effects in the Clinic
DARS: Dysphagia Aspiration Related Structure
