# Supplementary figures and images for: Intensity-modulated radiation therapy versus three-dimensional conformal radiotherapy in head and neck squamous cell carcinoma: long-term and mature outcomes of a prospective randomized trial
Source: Radiat Oncol. 2020 Sep 16;15:218. doi: 10.1186/s13014-020-01666-5 (PMC7493335; doi:10.1186/s13014-020-01666-5)

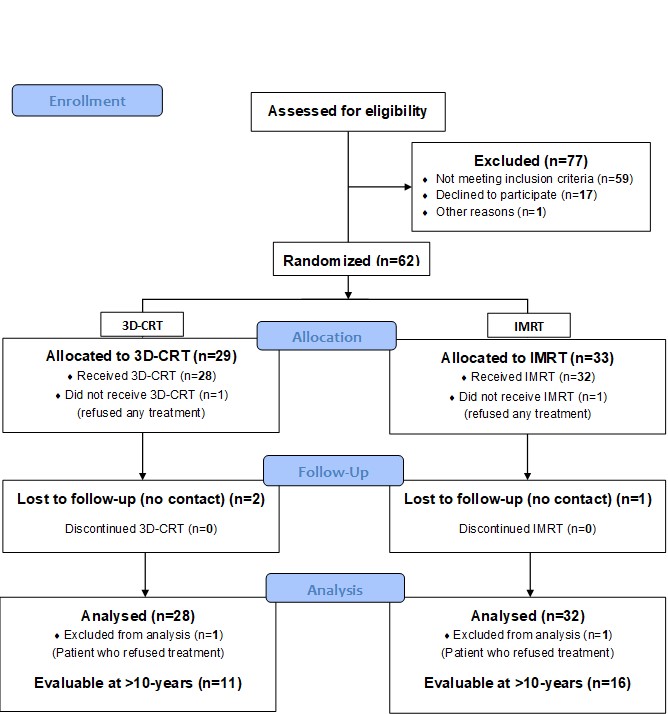

Supplement: Supplementary file 1 — Additional file 1 Supplementary Fig. 1 [file 13014_2020_1666_MOESM1_ESM.jpg]
